# Supplementary material for: Vesicular Zinc Modulates Cell Proliferation and Survival in the Developing Hippocampus
Source: Cells. 2023 Mar 11;12(6):880. doi: 10.3390/cells12060880 (PMC10047515; doi:10.3390/cells12060880)
Supplement: Supplementary file 1 [file cells-12-00880-s001.zip › cells-2280580-supplementary.pdf]

Supplemental Tables

Supplementary Table S1. Three-way ANOVA statistical results (effects of sex, genotype, and age).

**Cell Proliferation**	*df*	*F*	*p*
Sex	1, 60	0.26	.609
Genotype	1, 60	0.19	.664
Age	2, 60	4.71	**.013***
Sex × Genotype	1, 60	0.99	.323
Sex × Age	2, 60	5.95	**.004***
Genotype × Age	2, 60	6.03	**.004***
Sex × Genotype × Age	2, 60	1.47	.237
**Cell Survival**	*df*	*F*	*p*
Sex	1, 60	2.44	.124
Genotype	1, 60	27.34	**<.001***
Age	2, 60	130.10	**<.001***
Sex × Genotype	1, 60	0.09	.767
Sex × Age	2, 60	4.24	**.019***
Genotype × Age	2, 60	9.41	**<.001***
Sex × Genotype × Age	2, 60	2.25	.114
**Neuronal Differentiation**	*df*	*F*	*p*
Sex	1, 24	13.25	**.001***
Genotype	1, 24	0.19	.665
Age	2, 24	23.86	**<.001***
Sex × Genotype	1, 24	0.001	.972
Sex × Age	2, 24	0.36	.703
Genotype × Age	2, 24	2.29	.123
Sex × Genotype × Age	2, 24	0.48	.625

Note. df = degrees of freedom. $^*p < .05$.

Supplementary Table S2. Two-way ANOVA statistical results (effects of genotype and age) of BrdU⁺ cell quantification separated by sex and stage groups.

**Male**	*df*	*F*	*p*
**Cell Proliferation**			
Genotype	1, 30	1.11	.301
Age	2, 30	2.62	.089
Genotype × Age	2, 30	7.26	**.003***
**Cell Survival**			
Genotype	1, 30	14.20	**< .001***
Age	2, 30	54.30	**< .001***
Genotype × Age	2, 30	4.63	**.018***
**Female**	*df*	*F*	*p*
**Cell Proliferation**			
Genotype	1, 30	0.15	.705
Age	2, 30	7.66	**.002***
Genotype × Age	2, 30	0.73	.489
**Cell Survival**			
Genotype	1, 30	13.15	**.001***
Age	2, 30	82.14	**< .001***
Genotype × Age	2, 30	7.23	.003

Note. df = degrees of freedom. ***p* < .05**.

Supplementary Table S3. Shapiro-Wilk test of normality, skewness statistics, and kurtosis statistics for three-way ANOVAs (sex × genotype × stage) and two-way ANOVAs (genotype × age) of BrdU+ cell quantification separated by sex and developmental age groups.

**Male**	Shapiro-Wilk Test of Normality			Skewness Statistic	Kurtosis Statistic
	df	*W*	*p*		
**P6**					
WT Cell Proliferation	6	.97	.882	-0.69	0.74
WT Cell Survival	6	.94	.661	-0.96	0.49
KO Cell Proliferation	6	.95	.727	0.85	0.37
KO Cell Survival	6	.96	.822	0.09	-1.12
**P14**					
WT Cell Proliferation	6	.88	.279	1.24	0.93
WT Cell Survival	6	.78	**.040***	0.80	-1.86
KO Cell Proliferation	6	.91	.462	0.99	1.11
KO Cell Survival	6	.94	.628	-1.05	1.56
**P28**					
WT Cell Proliferation	6	.90	.370	1.01	0.18
WT Cell Survival	6	.99	.996	-0.27	0.17
KO Cell Proliferation	6	.95	.728	0.79	0.23
KO Cell Survival	6	.93	.584	0.92	1.51
**Female**	Shapiro-Wilk Test of Normality			Skewness Statistic	Kurtosis Statistic
	df	*W*	*p*		
**P6**					
WT Cell Proliferation	6	.96	.811	0.76	0.05
WT Cell Survival	6	.97	.873	0.24	-1.25
KO Cell Proliferation	6	.80	.062	1.49	1.46
KO Cell Survival	6	.79	**.043***	0.90	-1.63
**P14**					
WT Cell Proliferation	6	.89	.299	-0.11	-2.52
WT Cell Survival	6	.96	.809	0.38	1.01
KO Cell Proliferation	6	.90	.382	1.34	2.36
KO Cell Survival	6	.84	.141	1.28	0.91
**P28**					
WT Cell Proliferation	6	.85	.147	0.38	-2.30
WT Cell Survival	6	.90	.390	-0.90	-0.13
KO Cell Proliferation	6	.97	.883	-0.51	-0.57
KO Cell Survival	6	.95	.770	-0.11	-1.43

Note. n = 6 (per group). W = Shapiro-Wilk's statistic; df = degrees of freedom. **$^*p < .05$**. Although the assumption of normality was not met for some groups, factorial ANOVAs are moderately robust to violations of normality when sample sizes are equal. Given the equal sample size in each group and the skew and kurtosis values did not indicate a substantial departure from normality (i.e., within the range of ±2 and ±7, respectively), the ANOVAs were conducted under the assumption that normality was met.

Supplementary Table S4. Shapiro-Wilk test of normality, skewness statistics, and kurtosis statistics for three-way ANOVAs (sex × genotype × stage) of neuronal differentiation separated by sex and developmental age groups.

**Male**		Shapiro-Wilk Test of Normality			Skewness	Kurtosis
		df	*W*	*p*	Statistic	Statistic
**P6**						
	WT Neuronal Differentiation	3	.83	.180	-1.66	.
	KO Neuronal Differentiation	3	.75	**<.001***	-1.73	.
**P14**						
	WT Neuronal Differentiation	3	1.00	.956	0.12	.
	KO Neuronal Differentiation	3	.96	.637	-0.94	.
**P28**						
	WT Neuronal Differentiation	3	1.00	.895	-0.28	.
	KO Neuronal Differentiation	3	.92	.436	1.34	.
**Female**		Shapiro-Wilk Test of Normality			Skewness	Kurtosis
		df	*W*	*p*	Statistic	Statistic
**P6**						
	WT Neuronal Differentiation	3	.75	**<.001***	1.73	.
	KO Neuronal Differentiation	3	1.00	1.00	0.00	.
**P14**						
	WT Neuronal Differentiation	3	.75	**<.001***	1.73	.
	KO Neuronal Differentiation	3	.75	**<.001***	1.73	.
**P28**						
	WT Neuronal Differentiation	3	.	.	.	.
	KO Neuronal Differentiation	3	.80	.116	1.70	.

Note. n = 3 (per group). W = Shapiro-Wilk's statistic; df = degrees of freedom. $^*p < .05$. Although the assumption of normality was not met for some groups, factorial ANOVAs are moderately robust to violations of normality when sample sizes are equal. Given the equal sample size in each group and the skew values did not indicate a substantial departure from normality (i.e., within the range of ±2), the ANOVAs were conducted under the assumption that normality was met.

Supplementary Table S5. Levene's test of homogeneity of variance for three-way ANOVAs (effects of sex, genotype, and age).

	df	Statistic	*p*
Cell Proliferation	11, 60	2.63	**.008***
Cell Survival	11, 60	2.95	**.003***
Neuronal Differentiation	11, 24	3.24	**.008***

Note. Results are based on mean. *df*= degrees of freedom. **$^*p < .05$**. Although the assumption of homogeneity of variances was not met for some groups, factorial ANOVAs are moderately robust to violations of homogeneity of variance when sample sizes are equal, and the assumption of normality is not violated. Given the equal sample size in each group and the assumption of normality was met, the violations were considered minor and as a result, the ANOVAs were conducted under the assumption that homogeneity of variance was met.

Supplementary Table S6. Levene's test of homogeneity of variance for two-way ANOVAs (effects of genotype and age) separated by sex and stage groups.

**Male**	*df*	Statistic	*p*
Proliferation	5, 30	1.94	.117
Survival	5, 30	2.49	.053
**Female**	*df*	Statistic	*p*
Proliferation	5, 30	3.72	**.010***
Survival	5, 30	4.61	**.003***

Note. Results are based on mean. *df*= degrees of freedom. ******p* **< .05**. Although the assumption of homogeneity of variances was not met for some groups, factorial ANOVAs are moderately robust to violations of homogeneity of variance when sample sizes are equal, and the assumption of normality is not violated. Given the equal sample size in each group and the assumption of normality was met, the violations were considered minor and as a result, the ANOVAs were conducted under the assumption that homogeneity of variance was met.

Supplementary Table S7. Levene's test of homogeneity of variance for independent samples t-tests (effect of stage for male mice injected on P14) separated by genotype.

**WT**	Statistic	Significance	*t*	*df*	*p*
Equal variances assumed	9.31	**.012**	3.80	10	.003
Equal variances not assumed			3.80	5.13	**.012***
**ZnT3 KO**	Statistic	Significance	*t*	*df*	*p*
Equal variances assumed	0.95	.352	0.42	10	.682
Equal variances not assumed			0.42	8.01	.684

Note. Results are based on mean. *df*= degrees of freedom. ***p < .05**. When the assumption of homogeneity of variances was not met (**significance < .05**), the *t*, *df*, and *p* values from the "equal variances not assumed row" was used.

Supplementary Table S8. Additional statistical data of estimated number of BrdU+ cells (cell proliferation and cell survival) and percentage of BrdU+ cells colabelled with NeuN (neuronal differentiation, %) separated by sex and postnatal age groups. Statistics are reported as mean ± standard deviation.

Male	P6	P14	P28	Total
WT Cell Proliferation	2065.00 ± 594.69	2575.00 ± 1346.27	2331.00 ± 582.20	2323.67 ± 884.71
WT Cell Survival	938.00 ± 187.55	473.00 ± 152.71	299.00 ± 74.60	570.00 ± 309.64
WT Neuronal Differentiation	86.82% ± 2.45%	72.08% ± 2.89%	75.54% ± 7.32%	78.14% ± 7.85%
KO Proliferation	2858.00 ± 742.62	958.00 ± 452.06	2324.00 ± 689.54	2046.67 ± 1019.77
KO Survival	1340.00 ± 325.21	868.00 ± 261.36	263.00 ± 81.16	823.67 ± 508.79
KO Neuronal Differentiation	85.59% ± 4.17%	78.67% ± 6.11%	72.02% ± 3.54%	78.76% ± 7.17%
Proliferation	2461.50 ± 763.50	1766.50 ± 1276.65	2327.50 ± 608.45	2185.17 ± 951.32
Survival	1139.00 ± 328.84	670.50 ± 290.18	281.00 ± 76.66	696.83 ± 434.57
Neuronal Differentiation	86.21% ± 3.14%	75.37% ± 5.59%	73.78% ± 5.49%	78.45% ± 7.30%
**Female**	**P6**	**P14**	**P28**	**Total**
WT Proliferation	2350.00 ± 555.56	2497.00 ± 1084.81	1248.00 ± 296.48	2031.67 ± 889.77
WT Survival	906.00 ± 281.37	363.00 ± 104.18	267.00 ± 96.95	512.00 ± 336.23
WT Neuronal Differentiation	89.64% ± 2.04%	81.33% ± 2.31%	80.00% ± 0.00%	83.66% ± 4.78%
KO Proliferation	2862.00 ± 429.26	2171.00 ± 1571.80	1387.00 ± 328.97	2140.00 ± 1094.12
KO Survival	1465.00 ± 275.25	461.00 ± 148.69	289.00 ± 114.25	738.33 ± 563.38
KO Neuronal Differentiation	92.00% ± 4.00%	84.21% ± 0.36%	76.94% ± 9.59%	84.38% ± 8.34%
Proliferation	2606.00 ± 543.64	2334.00 ± 1298.80	1317.50 ± 307.27	2085.83 ± 984.38
Survival	1185.50 ± 394.52	412.00 ± 132.68	278.00 ± 101.67	625.17 ± 471.43
Neuronal Differentiation	90.82% ± 3.12%	82.77% ± 2.16	78.47% ± 6.29%	84.02% ± 6.60%
**Male + Female**	**P6**	**P14**	**P28**	**Total**
WT Proliferation	2208.50 ± 568.51	2536.00 ± 1166.37	1789.50 ± 716.87	2177.67 ± 886.92
KO Proliferation	2860.00 ± 578.30	1564.50 ± 1271.67	1855.50 ± 710.47	2093.33 ± 1043.46
Total Proliferation	2533.75 ± 652.37	2050.25 ± 1292.39	1822.50 ± 698.80	2135.50 ± 962.45
WT Survival	922.00 ± 228.59	418.00 ± 137.24	283.00 ± 84.15	541.00 ± 319.92
WT Neuronal Differentiation	88.23% ± 2.54%	76.71% ± 5.58%	77.77% ± 5.23%	80.90% ± 6.91%
KO Survival	1402.50 ± 294.57	664.50 ± 293.73	276.00 ± 95.45	781.00 ± 530.82
KO Neuronal Differentiation	88.79% ± 5.07%	81.44% ± 4.92%	74.48% ± 7.00%	81.57% ± 8.08%
Total Survival	1162.25 ± 355.98	541.25 ± 257.14	279.50 ± 88.07	661.00 ± 451.617
Total Neuronal Differentiation	88.51% ± 3.83%	79.07% ± 5.59%	76.13% ± 6.14%	81.24% ± 7.42%
